# In the shoes of junior doctors: a qualitative exploration of job performance using the job-demands resources model

**DOI:** 10.3389/fpsyg.2024.1412090

**Published:** 2024-10-24

**Authors:** Jia Long Chua, Zeenathnisa Mougammadou, Raymond Boon Tar Lim, Joshua Yi Min Tung, Gerald Gui Ren Sng

**Affiliations:** ^1^Preventive Medicine, National University Health System, National University Hospital, Singapore, Singapore; ^2^Saw Swee Hock School of Public Health, National University Health System, Singapore, Singapore; ^3^Department of Urology, Singapore General Hospital, Singapore, Singapore; ^4^Department of Endocrinology, Singapore General Hospital, Singapore, Singapore

**Keywords:** job demands—resources model, healthcare workers, junior doctor, well-being, job satisfaction

## Abstract

**Background:**

This qualitative study aimed to explore the factors affecting job performance amongst junior doctors working for public healthcare institutions in Singapore. Within these institutions, junior doctors experience challenges with maintaining a balance in job demands and resources, leading to strain. Exploring the lived experiences of these junior doctors is essential when reviewing workplace and organizational factors that contribute to stress on an individual level, providing valuable insights to address these challenges effectively.

**Method:**

Semi-structured interviews were conducted with 20 junior doctors in Singapore, ranging from house officers to senior residents. Framework analysis was performed on transcribed de-identified interviews to identify themes deductively based on the Job Demands-Resources (JD-R) Model.

**Results:**

Themes were identified and contextualized based on the exiting JD-R model. These themes shed light on how work demands, resources and personal factors influence the job performance of junior doctors and job satisfaction.

**Conclusion:**

The study offers valuable insights into the specific issues disrupting the job demands and resource balance in Singapore Public Healthcare Institutions and their correlation with job performance. Our data suggests that job performance may be associated with job satisfaction. By understanding these factors, targeted efforts can be developed to improve working conditions for junior doctors, fostering their growth and engagement within the public healthcare system.

## 1 Introduction

Perceived job performance and job satisfaction are closely linked, with each influencing the other. Job satisfaction enhances motivation, engagement, and commitment, leading to better perceived performance (Lu et al., 2017). In fact, the lack of job satisfaction can lead to decreased dedication to the organization, diminished job effectiveness, and ultimately, attrition of workers from the workforce (Ray, 2021; Faragher et al., 2005). Conversely, perceived high job performance boosts job satisfaction by fostering a sense of accomplishment and pride. This cycle creates a feedback loop where satisfaction and performance mutually enhance each other. Understanding and fostering this dynamic is crucial for organizations to improve both employee satisfaction and perceived job performance.

In the healthcare sector, Junior Doctors (JDs), are qualified doctors working in public healthcare institutions while awaiting or undergoing postgraduate training who play a vital role in service delivery in Singapore. However, recent studies have reported lower job satisfaction scores among JDs in Singapore, on a backdrop of decreasing job satisfaction globally amongst healthcare professionals such as General Practitioners (Tung et al., 2023; Chang et al., 2017; Deng et al., 2024). While factors like long working hours are commonly cited contributors to lower satisfaction, it is essential to delve deeper into other potential determinants (Alrawashdeh et al., 2021). The demanding nature of their roles not only induces stress but also leads to imbalanced work-life arrangements, financial anxieties, and negative health behaviors, impacting perceived work performance and satisfaction (Wainwright et al., 2017).

Despite extensive research on stress and burnout among medical professionals globally, most studies have focused on European contexts which does not address the unique cultural and healthcare system intricacies present in Singapore (Wainwright et al., 2022; Riley et al., 2021). In Singapore’s public healthcare sector, JDs represent a significant proportion of the doctor workforce. Upon completing medical school, they undergo a year of training as a house officer, followed by either residency or specialisation training, or they remain as medical officers. Additionally, JDs who graduate from local medical schools are typically bonded to serve in public healthcare institutions for 5 years, limiting their employment mobility (‘Review of Junior Doctors’ Work Hours among Steps to Improve Healthcare Workers’ Well-Being’, 2022). This structural context, combined with the cultural expectations and hierarchical nature of the Singaporean healthcare system, creates unique challenges and stressors for JDs, which may differ significantly from those faced by their European counterparts.

This study marks the first qualitative exploration into the factors influencing job satisfaction among JDs in Singapore’s public healthcare institutions. Within these settings, JDs navigate a myriad of personal, interpersonal, and organizational dynamics that frequently culminate in stress, impacting their mental well-being and overall job satisfaction. Understanding their experiences is crucial for identifying and addressing workplace stressors effectively.

To explore the relationship between job demands, resources, work engagement, and job satisfaction, this study employs Bakker and Demerouti’s Job Demand-Resources (JD-R) model (Bakker and Demerouti, 2017, 2018). This model stands out over other theories for three main reasons. First, the JD-R model integrates both demands and resources, providing a balanced view of the factors that affect work engagement and performance. This dual focus is crucial in understanding the complete picture of JDs’ work environments. According to the JD-R model, job demands refer to the physical, psychological, social, or organizational aspects of the job that require sustained effort and are associated with physiological and psychological costs. Conversely, job resources refer to the physical, psychological, social, or organizational aspects of the job that help achieve work goals, reduce job demands, and stimulate personal growth and development. Personal resources are components of the self that are linked to resiliency and self-perceived control and influence over their work environment, which is postulated to mediate the relationship between job resources and engagement and influence the perception of job resources (Xanthopoulou et al., 2007). The model suggests that high job demands can lead to job strain and impaired well-being, whereas high job resources foster work engagement and motivation. When job resources are sufficient, they can buffer the impact of job demands on job strain, leading to positive outcomes such as perceived work performance and job satisfaction. Conversely, insufficient job resources can exacerbate the negative effects of job demands, adding to a cycle of increased strain, reduced motivation, and declining job performance.

Second, this model posits that job performance depends on the interplay between job and personal resources and job demands. It is adaptable to various work environments and can be tailored to specific occupations and contexts. This flexibility makes it an ideal choice for studying JDs, whose roles can potentially vary across different healthcare settings. Third, the JD-R model has been extensively validated in various professional settings, including healthcare. In the realm of physicians, JD-R model has previously been leveraged to explore burnout, well-being (Jing et al., 2023; R. Scheepers et al., 2020) and has been successfully applied in quantitative models to predict intention to leave (Chênevert et al., 2021; Maniscalco et al., 2024). Cultural context, including societal expectations, work culture and work-life balance, significantly influences JDs’ roles (Ng et al., 2018; Leu et al., 2023). Additionally, unique job demands and resources, patient care dynamics, and expectations further shape JDs’ experiences. These factors underscore the need for a detailed examination of how specific elements of the Singaporean context influence the perceived job performance and satisfaction of JDs, providing a comprehensive and contextually relevant analysis distinct from existing international studies. Therefore, this study aims to explore the unique job demands and resources, including personal resources, as well as cultural and societal factors, that collectively influence job performance and satisfaction using the JD-R model among junior doctors in Singapore.

## 2 Methods

Semi-structured interviews using an open-ended guide with specific prompts were conducted to explore participants’ physical (such as sleep, diet, exercise) and mental (including stress, job satisfaction, social aspects) well-being based on the JD-R model (Supplementary material). Invitations for interviews were sent via institutional email to all JDs in Singapore from July to December 2021. JDs were defined as physicians in training or those awaiting postgraduate training in public healthcare institutions. Interested JDs were contacted for Zoom interviews scheduled at their convenience. Of the 21 JDs who showed interest, 20 gave written consent and participated in virtual interviews, consenting to audio recordings. Interviews lasted 60–90 min.

All interviews were transcribed verbatim by the research team (JL, ZA) for analysis. To safeguard the anonymity of participants and patients, gender-neutral pronouns and details related to incidents were altered to maintain privacy. The study was given full ethics approval by the National University of Singapore Saw Swee Hock School of Public Health (NUS-IRB-2022-248).

### 2.1 Analysis

The data were analyzed using framework analysis (Brooks et al., 2015) through a deductive approach, anchored in the theoretical constructs of the Job Demands-Resources (JD-R) Model (Gale et al., 2013; Brooks et al., 2015). The choice of a deductive approach was informed by the study’s objective to examine how the pre-defined dimensions of the JD-R model—job demands, job resources, and personal resources—manifest within the context of JDs’ experiences. While qualitative research traditionally favors an inductive approach to allow new themes to emerge, this study required a more theory-driven analysis to systematically test and refine an existing model within a specific context. The deductive framework allowed for structured analysis of key theoretical constructs, while also providing the flexibility to explore deviations or extensions to the JD-R model. This approach not only ensures the robustness of our findings but also offers valuable insights into how the JD-R model can be contextualized for different professional groups.

The transcripts were read to gain an initial understanding of the complexity in the data collected. Meetings between all researchers (JL, ZA, JT, GS) facilitated discussion of the concepts and themes that arose from the data to enable refinement of ideas and achieve consensus on the final set of themes. The themes were then applied to the existing JD-R model with consensus from all authors, a deviation from the usual framework analysis process which involves the development of a working framework or model first. The data was then charted and interpreted by JL and ZA before development of the final contextualized JD-R model. Anonymized quotations from the interviews are included to provide additional context (Supplementary material).

## 3 Results

### 3.1 Participant characteristics

Participants included 9 women and 11 men, aged 27–36 years of age, employed across Singapore Public Healthcare Institutions (see Table 1). The participant group was spread across the varying spectrum of JDs in Singapore which comprised House Officers (HOs), Medical Officers (MOs), Residents (which may either be House or Medical officers) and Senior Residents. Most participants had received their medical degree in Singapore, and all were working in public hospitals in Singapore at the time of the interview (see Table 1).

**Table 1:** Demographic of participants.

Number of interviewees	*N* = 20
Mean age (range)	27.9 (25–35)
Local/overseas medical education	75% (local) / 25% (overseas)
Appointment^1^	House officer (*n* = 6), Medical Officer (*n* = 10), Resident (*n* = 2), Senior Resident (*n* = 2)
Years of working experience, mean	2.95

^1^As Singapore’s healthcare system is based on that of the United Kingdom, the description of job grades are as follows: House Officers (PGY1), Medical Officers (PGY2 and above, but not in residency), Residents (PGY2 and above and in formal training), Senior Residents/Registrars (Fellows).

### 3.2 Summary of themes

The qualitative study explored the various stressors experienced by JDs, identifying 8 overarching themes based on the JD-R model—namely, lack of job resources, personal resources affect motivation of JDs, increased in job demands, motivation affects perceived job performance, increased strain in JDs, limited job-crafting, self-undermining and perceived work performance (Table 2).

**Table 2:** Summary of themes and key definitions.

Themes/definition	Definition of subthemes
**Theme 1: Lack of job resources** Job resources encompass the physical, social, financial, or organizational aspects of a job that aid in achieving work-related outcomes while mitigating the impact of job demands on individuals. A deficiency in resources to address heightened demands can lead to a demand-resource imbalance.	**Subtheme 1.1: Lack of adequate manpower to cope with increased demand** Inadequate manpower refers to an insufficient number of doctors in a team, hindering effective care delivery, ultimately leading to an imbalance of job demands and resources.
	**Subtheme 1.2: Lack of effective organizational and financial initiatives to cope with increased demand or its consequences** Ineffective organizational programs, policies, and financial compensations fail to yield positive outcomes for JDs and do not mitigate the physical and psychological consequences of increased job demands.
	**Subtheme 1.3: Hierarchical nature of the healthcare profession** The healthcare profession’s hierarchical nature denotes its structured organization, with varying levels of authority and responsibility among roles like consultants, residents, house officers, nurses, and support staff. This structure shapes communication, decision-making, and opportunities for professional growth.
	**Subtheme 1.4: Lack of training opportunities** Training opportunities refers to specialist or residency training positions. JDs would be required to demonstrate the necessary professionalism, ethics, and attitudes in their work to successfully compete for such positions.
	**Subtheme 1.5: Lack of supportive actions by colleagues and supervisors** The absence of supportive actions, such as proactive assistance, workload management, emotional support, and mentorship from colleagues and supervisors, can lead to an imbalance in job demands and resources. Without this support, employees may struggle to cope with high job demands more likely to experience increased strain, reduced motivation and reduced satisfaction and engagement
**Theme 2: Personal resources impacts motivation of JDs** Personal resources encompass the perceived ability to fulfil work duties and achieve outcomes, organizational-based self-esteem fostering a sense of value within the organization, and optimism nurturing a positive outlook. These factors enhance motivation, positively impacting work engagement, and mitigating the adverse effects of strain on work engagement.	**Subtheme 2.1: Perceived capability to fulfil responsibilities in an effective manner** Perceived capability to respond to job demands, execute tasks, and handle challenges effectively is a crucial personal resource. This perception influences how employees view and react to job demands and instil confidence in managing responsibilities and overcoming obstacles.
	**Subtheme 2.2: Innate optimism or personal demand** A positive outlook on life and an inherent tendency to expect favorable outcomes. Is a psychological trait characterized by a general expectation that good things will happen and that challenges can be overcome. It can be influenced by factors such as faith, religion, and spirituality. The concept of personal demands relates to requirements that JDs set for their own individual performance that necessitates investment of time and effort and potentially associated with stress.
**Theme 3: Increase in job demands** Job demands necessitates the continual application of physical, cognitive, and emotional efforts or skills resulting in the psychological, cognitive, or physical consequence.	**Subtheme 3.1: Long working hours** JDs perceive long working hours as exceeding their regular schedules, demanding sustained physical, cognitive, and emotional exertion.
	**Subtheme 3.2: Emotional load from direct patient care** Emotional load involves exposure to distressing or challenging patient encounters or work situations.
	**Subtheme 3.3: Cognitive load (from pre-rounding, administrative duties, and high professional standards)** 3.3.1 Pre-rounding Pre-rounding involves the review and examination of each patient before ward rounds and requires JDs to arrive early for work and adds to their list of duties. 3.3.2 Administrative tasks Administrative tasks like meeting academic teaching obligations, which, while not directly tied to clinical patient care, add to their workload alongside their clinical responsibilities. 3.3.3 High professional standards JDs are held to high standards in quality, ethical conduct, continuous improvement, and excellence in care delivery. These standards can significantly impact their work engagement and commitment, either positively by fostering pride in their work and a commitment to delivering excellence, or negatively by adding pressure and stress.
**Theme 4: Motivation improves the perceived job performance** Motivation drives workers to be engaged with their work and promotes job performance and satisfaction. Job resources, both intrinsic and extrinsic, enhance work engagement, promote exceptional performance, and facilitate goal achievement. Whereas, personal resources, intrinsic to individuals, positively motivate and impact work engagement by instilling a sense of fulfilment and importance in their tasks, aligning with work goals.	**Subtheme 4.1: Increased work engagement** Motivated work engagement represents the depth of emotional involvement, enthusiasm, and dedication JDs exhibit towards their tasks, fuelled by intrinsic and extrinsic motivations. Engaged JDs demonstrate a profound connection to their work, driven by a sense of purpose and fulfilment derived from aligning with their work goals and organizational values. This state of engagement is shaped by factors such as individual motivation, supportive work culture, and the balance between job demands and resources.
	**Subtheme 4.2: Commitment to learning and professional growth** This aspect of work engagement involves seeking out and embracing opportunities for development, such as training programs, educational courses, on-the-job learning experiences, and feedback from colleagues and supervisors to perform better at work.
T**heme 5: Increased strain on JDs** Strain is defined as negative consequences that arise due to the imbalance between job demands and resources. Strain can be further classified into job and non-occupational strain.	**Subtheme 5.1: Increased job strain** Job strain, in the context of healthcare workers, refers to increased physical, cognitive, and emotional exhaustion leading to lack of empathy and fatigue, impacting work engagement.
	**Subtheme 5.2: Increased non occupational strain due to lack of social care and self care activities** Non occupational strain in the context of healthcare workers, refers to negative consequences outside of the work environment and job scope such as lack of time for self-care activities. Lack of self-care activities refers to the social time with family and friends, and good lifestyle habits such as time to exercise, eat healthy and pursue hobbies which ultimately impact performance and engagement at work.
**Theme 6: Job crafting** Job crafting refers to the process where employees actively make changes in their work tasks, working relationships and perception of work to improve job resources and reduce undesirable or hindrance demands.	**Subtheme 6.1: Limited job crafting opportunities for JDs** Task crafting requires organizational support to enable JDs to adjust the available job resources to as to align better with their skills and interests and tackle the job demands better. Limited job crafting is associated to decreased motivation impacting perceived job performance and job satisfaction
	**Subtheme 6.2: Relational crafting** Relational crafting relates to the building of supportive networks by JDs with other peers and healthcare workers with the intent of creating positive or effective interpersonal relationships.
**Theme 7: Self-undermining** Self-undermining refers to behaviours that create obstacles, which may lead to higher levels of job demands and even higher levels of job strain.	**Subtheme 7.1: Perceived conflict** Engaging or avoiding resolution of conflict can create hostile work environments, leading to decreased support and resources and increased strain.
	**Subtheme 7.2: Burnout** Burnout refers to a state of work-related state of exhaustion, apathy, and sense of cynicism, leading to a cycle of increased strain and decrease in job performance.
**Theme 8: Perceived work performance** Perceived work performance refers to JDs evaluate their effectiveness and efficiency in fulfilling their job responsibilities. This self-assessment is closely linked to their level of job satisfaction, which reflects their contentment with their work. Work satisfaction, or job satisfaction, indicates employees’ level of contentment with their job. This satisfaction is closely linked to work engagement, as higher satisfaction often corresponds to increased engagement. Conversely, heightened engagement frequently leads to greater job satisfaction. This reciprocal connection underscores the interdependence between these two constructs in shaping employees’ experiences and organizational outcomes.	

#### Theme 1: Lack of job resources

The following findings illustrate how the challenges faced by JDs in Singapore, specifically related to manpower shortages and organizational response, contribute to an imbalance in job demands and resources. These factors significantly affect their job satisfaction and well-being. The lack of adequate manpower intensifies job demands, increasing strain and reducing satisfaction. Furthermore, the ineffective organizational response to feedback and mental well-being programs exacerbates this imbalance, as the JDs lack the necessary job resources to cope with their high demands. While, the lack of training opportunities, work structure and support received during work also exacerbates the demand-resource imbalance.

##### 1.1 Lack of adequate manpower to cope with increased demand

*“It becomes very stressful when there’s a lack of manpower … those were the times where I was most stressed … because there are so many patients for me to take care of, it’s not that I cannot take care of all of them but like, you cannot give them to me all at the same time.”* (Participant 6, Medical Officer)

JDs reported manpower challenges in hospitals, which intensified the workload for their colleagues during periods of absence. These absences were categorized as either work-related, such as post-overnight call recuperation or attendance at work-related training, or non-work-related, including overseas leave or medical leave. The increased job demands and the lack of manpower to manage it often increased the job strain amongst JDs. The demand-resource imbalance also affected their job performance and satisfaction as they were not able to complete the job tasks efficiently and effectively.

##### 1.2 Lack of effective organizational and financial initiatives to cope with increased demand or its consequences

*“But ultimately, if I feel like, no matter how high I escalate the issues, that if I tried to give constructive feedback … everything just falls on deaf ears … then I do not see what role I can play in that workplace anymore.”* (Participant 4, Medical Officer)

JDs reported that their feedback often did not result in changes to work conditions, leading to dissatisfaction with feedback management and a lack of visible outcomes. Some were hesitant to provide feedback regarding concerns with job demands or resources, due to fears of repercussions or scrutiny. The absence of transparent, anonymous, and effective feedback mechanisms contributed to the imbalance between job demands and resources, increasing JDs’ work strain. Additionally, while mental well-being programs were implemented by organizations, JDs found them ineffective due to a lack of customisation to their specific work situations. Program scheduling during busy periods, like lunchtimes, prevented JDs from participating, limiting the benefits they could gain from these well-being initiatives.

*“I think the pay is actually bad in view of the massive loan that I had to take out, I do feel financially stressed up because most of the pay even if it’s sufficient goes towards repaying the loan so at the end of the day I’m still kind of like not the most financially well off in terms of my peers at where it started working.”* (Participant 10, House Officer)

While JDs are well-remunerated compared to the median income in Singapore, many see their remuneration as low for the job demands they face, especially compared to private healthcare or other professional sectors. In addition, the competitive nature of specialist training selection and the mandatory 5-year bond for locally trained doctors impact their motivation and career choices. Of note, some who did not wish to enrol into a residency programme reported less motivation to work beyond their bond obligations.

##### 1.3 Hierarchical nature of the healthcare profession

*“It’s very different from Ireland (alma mater), so I think in Singapore the culture shock … (comes from) this clear hierarchy of consultants, ACs (associate consultants), Registrars, MOs, and more HOs around it. Yeah, it’s a bit of a shock to me that the consultant is this king that walks through the wards and you present the cases as they walk by.”* (Participant 17, House Officer)

JDs, particularly those who received their medical education overseas, observed distinct differences in the organization of clinical teams in Singapore compared to their experiences abroad. The hierarchical structure of medical teams in Singapore created a perceived divide between consultants and JDs. The pronounced hierarchy often led to feelings of distance and formality in interactions with senior medical staff, impacting the psychological strain and motivation to work.

##### 1.4 Lack of training opportunities

*“You are just kind of like waiting for time to go by until you can leave if you are not specialising then for those that are specialising it’s like even more stressful honestly, because it has become so competitive (to secure training positions)”* (Participant 16, Medical Officer).

JDs interested in specialist training opportunities experienced significant stress due to the highly competitive nature of these positions, resulting in a pressing need to excel in their clinical postings. Conversely, the limited availability of training opportunities and resources led some JDs to feel less pressure to perform well at work, potentially resulting in decreased motivation.

##### 1.5 Lack of supportive actions by colleagues and supervisors

*“When it gets down to it, your friend (colleagues) will help you … I felt supported by that many times.”* (Participant 1, Medical Officer)

While most JDs highlighted a lack of resources, a discordant subtheme stemmed from the positive supportive actions by peers and colleagues, which expanded the job resources of JDs especially during challenging situations. Examples of this would include open communication, provision of psychological support services, and fostering a culture of collaboration, respect, and work-life balance. Many JDs cited positive experiences interacting with a direct supervisor who were mindful of their workload and altruistically aided. These JDs shared their gratitude for the support received and highlighted similar experiences where they had tried to mirror this action, potentially indicating a greater commitment to work.

#### Theme 2: Personal resources impacts motivation of JD

##### 2.1 Perceived capability to fulfil responsibilities in an effective manner

*“I think to some extent, it does, because with experience you kind of know what common mistakes you have made, and you know what to look out for. If you know you are tired and you know, you are doing something where you might make a mistake, I think, in a sense, you can kind of remind yourself, consciously to look out for the mistake that you usually make when you are tired. But I think you are still as prone to errors, it’s just that your checking system is a bit better with experience.”* (Participant 11, House Officer)

JDs develop greater self-efficacy with working experience, which is a critical determinant of personal resources as defined by the Job Demands-Resources (JD-R) model. Self-efficacy refers to the belief in one’s capability to execute necessary actions to achieve specific performance goals. As JDs gain experience, they become more adept at recognising and mitigating common mistakes, especially under fatigue. This enhanced ability to anticipate and correct potential errors reflects their growing confidence and competence in managing job demands. In the JD-R model, personal resources like self-efficacy play a pivotal role in balancing job demands and resources, enabling JDs to navigate their complex roles with increased confidence, potentially contributing to increased motivation at work, and eventually perhaps resulting in increased perceived job performance.

##### 2.2 Innate optimism or personal demand

*But at least for me, I do not feel that’s the case, I think there’s more about things in life than excelling just at work. I think, building yourself as a person is more important. So, I guess it’s really all about priorities but some people of course, really see work as everything so I’m sure for those people things will be a lot more difficult, because you will then expect yourself to be at your best all the time*. (Participant 14, Medical Officer)

JDs personal resources are closely tied to the optimism of their outlook. An optimistic perspective allows JDs to prioritise personal growth and well-being alongside their professional responsibilities. This positive outlook helps them navigate the challenges of their demanding roles with a balanced approach, recognising the importance of self-development beyond just excelling at work. For those who maintain an optimistic view, it becomes easier to manage stress and remain motivated, as they do not tie their self-worth solely to their professional performance. Conversely, JDs who view work as their primary focus may struggle more, facing greater difficulty and stress as they strived to meet high expectations continuously. Thus, innate optimism plays a crucial role in enhancing personal resources, enabling JDs to sustain their motivation in a challenging healthcare environment.

The concept of self-expectations aligns with the idea of personal demands, where JDs impose expectations on their clinical performance, requiring commitment and energy to achieve these goals. These personal demands may act as double-edged swords, driving JDs in pursuit of higher performance while potentially generating greater stress and anxiety in the face of setbacks.

#### Theme 3: Increase in job demands

Increased work demands for JDs in Singapore manifest through long working hours, heightened cognitive and emotional loads, and extensive non-clinical administrative tasks, contributing to job strain and reduced satisfaction. Extended shifts, including 24-h on-calls, lead to significant fatigue and disrupt sleep patterns, impacting patient care. As JDs progress, they assume greater responsibilities and face emotionally challenging situations, further exacerbating strain. Balancing clinical duties with mandatory Continuing Medical Education (CME) sessions reduces their control over schedules, increasing overall job strain. These factors collectively underscore the multifaceted impact of work demands on JDs’ perception of their work demand-resource balance.

##### 3.1 Long working hours

*“Two main things that will lead to feeling a lot more tired or burnt out, the first one is calls because everybody hates calls, and it’s 30 plus hours of non-stop work.”* (Participant 8, House Officer)

JDs in Singapore experience a demanding work schedule marked by long hours from patient care as well as pre-rounding requirements in addition to weekend ward rounds and on-call duties which could take the form of a 24-h call or a consecutive duration of 12-h duties known as the ‘float’ system where doctors would be assigned to either a day or night shift. In either system, JDs faced issues with acclimatising to the sudden changes in sleep patterns, especially those who were on night shifts resulting in increased fatigue and subsequently resulting in job and non-occupational strain. Ultimately, this had a negative impact on job satisfaction.

*“I’m completely exhausted, my brain is functioning at 20% of capacity yet I’m making life-or-death decisions on call or like post-call the next morning. So, I’m literally falling asleep at the computer… How can I provide the care that I cannot get access to?”* (Participant 4, Medical Officer)

Many JDs reported that fatigue adversely impacted their ability to provide optimal patient care, with feelings of sleepiness and exhaustion being common. Some JDs expressed guilt for deferring non-emergent requests, emphasising the need to prioritise rest to ensure functionality for emergent cases. While most did not report direct patient errors attributable to fatigue, several JDs recounted near-miss incidents, such as dosing errors that were intercepted by pharmacists before reaching patients.

##### 3.2 Emotional load from direct patient care

*“When I was a HO, one patient’s son made me cry … he was just being very unreasonable and accusing me of stuff that wasn’t true”* (Participant 3, Medical Officer).

As JDs progressed from house officers to medical officers (MOs), they took on more patient care responsibilities, including assisting in surgeries, reviewing patients in clinics, and preparing for multidisciplinary discussions. They also frequently managed emotionally challenging situations, such as witnessing patient suffering, delivering difficult news, or facing patients’ and families’ high expectations. Many JDs had cited past experiences dealing with unhappy patients and family members, feeling unsupported. Continuous exposure to these stressors had led to emotional exhaustion in some, resulting in increased job strain and decreased motivation to perform at work.

##### 3.3 Cognitive load (from pre-rounding, administrative duties and high professional standard)

###### 3.3.1 Pre-rounding

*“I mean officially you are not supposed to (pre-round) but let us be honest, it happened, and it happens.”* (Participant 18, Medical Officer)

Many JDs, particularly those trained overseas, noted pre-rounding as a significant difference in their work culture compared to their training in countries like the United Kingdom and Australia. Pre-rounding, which involves assessing patients before ward rounds, increased the efficiency of ward rounds by reducing the time taken for clinical assessment by inpatient teams, at the cost of longer working hours and workload for JDs highlighting a trade-off between efficiency and workload in healthcare.

A minority of JDs view pre-rounding as essential for patient care, as it aids consultants who rely on notes from pre-clerking for patient progress and decision-making. Eliminating pre-rounds would necessitate a shift in work culture, requiring more time for ward rounds and greater involvement from attending physicians in clinical data interpretation and decision-making, potentially reducing the time available for surgical procedures and outpatient consultations.

###### 3.3.2 Adminstrative tasks

*“When you finish rounding, you have a lot of changes to do and that often runs into the time when you are supposed to go for teaching.”* (Participant 10, House Officer)

Apart from providing clinical care, JDs are expected to balance multiple tasks during a workday which reduces their perception of control over their time and schedule. Specifically, the obligation to attend Continuing Medical Education (CME) talks, as mandated by their respective departments, was frequently mentioned as a contributing factor to their increased work demands leading to job strain.

###### 3.3.3 High professional standards

*“After I made that one mistake, he (consultant) kept going after, like, all the small details to the point where I feel like, every morning when I present I have to back up what I said, like, Oh, it is here, or I saw this here, and then show it to him so that he does not like doubt what I’m saying.”* (Participant 11, House Officer).

The medical profession maintains high standards of professional conduct, yet JDs frequently face pressure to consistently deliver exceptional performance to avoid suspicion from senior colleagues. Practicing medicine under the pervasive fear or anxiety about their clinical competence exacerbates the existing job demands and resources balance. This heightened pressure significantly influences their perception of workplace strain and may negatively affect their ability to achieve job satisfaction.

#### Theme 4: Motivation improves the perceived job performance

Motivation in the workplace is a multifaceted concept that drives employee engagement, performance, and satisfaction. Motivations may arise as a natural extension of job and personal resources, channeled, and perceived positively by JDs, forming extrinsic and intrinsic motivations, respectively.

##### 4.1 Increased work engagement

*“It’s a bit mixed but overall, it’s more positive, I genuinely really enjoy being a doctor, I enjoyed the role of what it entails to be a doctor, of what we try to do within a community, I enjoy the roles where I get to interact directly with patients.* (Participant 12, Resident)

Despite increasing work demands, some JDs still demonstrated strong work engagement exhibited by the depth of their emotional involvement with work. This engagement stemmed from their aspirations and commitment to the medical profession, bolstered by extrinsic factors such as positive work cultures, professional recognition as well as intrinsic factors such as their personal relationships and spirituality.

“*Working as a junior for a while. You also feel like you are very, very replaceable. And I guess that also like makes you feel… Like your work, I do not know. It’s not very meaningful. Well, like you are not very meaningful.”* (Participant 3, Medical Officer)

Conversely, many JDs highlighted instances where they did not feel valued or appreciated by the organization or peers within the public healthcare system with only a minority of JDs who had past experiences where patients or other healthcare professionals demonstrated overt appreciation for their work. This lack of recognition negatively impacted their work engagement and, for some, diminished their desire to pursue further professional training. Feelings of under-appreciation contributed to a sense of replaceability and insignificance, adversely affecting their overall job satisfaction and commitment to their roles.

##### 4.2 Commitment to learning and professional growth

*“At the end of the day, you must accept that you are still human and mistakes must be made and can be made. You must face the consequences for them, of course, but part of it is realising that you are never your best all the time … for me least I take it as a learning opportunity”* (Participant 1, House Officer).

Many JDs were acutely aware of the challenges in patient care and were introspective about their own performance and potential for individual growth. Despite the challenges posed by job demands and limited resources, JDs adopted a positive approach to these situations, demonstrating values of dedication and a hope to better contribute to patient care. This ability to find positivity and value in their work significantly contributed to their overall job satisfaction and engagement.

*“I think for me, at least I have bosses who care, and they do keep in touch here and then… That’s one of the things that that drives me to do my work every day. And I enjoy teaching so the medical students that I’ve seen, grown from year three, year four, year five, all the way to HO/MO”* (Participant 14, Medical officer).

JDs reported that the interpersonal bonds formed with senior colleagues who valued education were a significant motivator for their continued engagement in the profession. These meaningful mentoring relationships not only supported their professional development but also inspired JDs to provide similar educational experiences for medical students. This reciprocal teaching and mentoring dynamic foster a supportive learning environment and enhances overall job satisfaction and work engagement.

#### Theme 5: Increased strain on JDs

Strain in the workplace encompasses both job-related and non-occupational factors that negatively impact the well-being and job satisfaction of employees. For JDs in Singapore, increased job strain arises from the demanding nature of their work, characterized by long hours, high cognitive and emotional loads, and the pressure to perform. This strain is compounded by non-occupational factors such as the inability to maintain a work-life balance and engage in self-care activities due to rigid schedules and academic responsibilities. Together, these elements contribute to heightened stress levels, emotional fatigue, and a decrease in overall job satisfaction, illustrating the multifaceted nature of strain experienced by JDs.

##### 5.1 Increased job strain

*“The main barrier towards stress management is that you have too much work… but when you ask how I still try to manage my stress better it would really just be like to have more rest, which I cannot.”* (Participant 16, Medical Officer).

Strain is defined as negative consequences that arise due to the imbalance between job demands and resources. Work culture and motivation also impacts the relationship strain can be further classified as job and non-occupational strain. JDs experienced increased physical and emotional fatigue primarily from long working hours, leading to heightened irritability and negative changes in their demeanor, which strained dynamics amongst healthcare staff. Additional stress resulted from care coordination responsibilities and fear of not meeting expectations. The increased physical and emotional fatigue and stress often leads to lack of empathy amongst JDs and is exacerbated by the protocolized task-based work JDs undertake, risking patient dehumanization and further reducing work engagement.

On the other hand, JDs who have entered a residency training programme often feel a significant amount of pressure to perform well in their rotations while devoting sufficient time to study for postgraduate examinations. The fear of failing required postings or examinations and stalling in their professional development can contribute to a cycle of stress, increasing the job strain and reducing job satisfaction.

##### 5.2 Increased non occupational strain due to lack of social care and self care activities

*“For me, doing some kind of exercise is very helpful and once your day ends late, that outlet or that window of time is taken away”* (Participant 18, Medical Officer)

JDs often have limited flexibility in adjusting their work schedules for personal commitments due to long working hours, varying call, or work schedules as well as the presence of academic responsibilities (e.g., studying for postgraduate examinations as well as research work). This lack of autonomy can make it challenging to engage in social or self-care activities. Notably, a considerable number of JDs reported difficulties in maintaining physical exercise routines and making healthy dietary choices, often resorting to readily available fast food to manage time constraints and decompress from work pressures. This restricted ability to prioritise self-care exacerbates the effect of job demands and resource mismatches on JDs, further reducing their work engagement.

#### Theme 6: Job crafting

##### 6.1 Limited job crafting opportunities for JDs

*“There’s a lot of things you want to say and cannot because we are bonded cannot afford to lose our jobs, we cannot afford to lose the money, many of us are still trying to get residency so we cannot afford to lose our career. So, you cannot speak up it’s made difficult. So anonymously feedback channels would be great.”* (Participant 4, Medical Officer)

*“So, the stress comes from how I want to do it (communicating with families and patients), the system does not recognise it, over the long time, when I try to do it at my own expense, with my own time, I become burnt out. So, I decided that I had to rebalance my priorities and choose something that allows me to do it.”* (Participant 19, Senior Resident).

JDs reported challenges in task crafting, citing difficulties in negotiating changes in work condition changes or additional job resources with supervisors or hospital management. This was particularly challenging given their bonded status, financial dependencies, and aspirations for residency, which collectively hindered their willingness to voice concerns or request modifications. To adjust their expectations and align their values to optimize perceived job performance, one participant chose to commit to a certain specialty that would allow them to practice medicine in a way which prioritized patient communication. Such efforts undertaken by JDs, highlighted the lengths they would have to take in order to achieve job crafting in an healthcare institution.

##### 6.2 Relational crafting

*“Oh, I see the other nurse ask, “help me turn the patient?” I say no problem, I help them… I think all these small things do help. So, they feel like, okay, this doctor is helping me”* (Participant 18, Medical officer).

In contrast, JDs frequently engaged in relational crafting to improve their work environment and available resources. For example, by assisting colleagues and building reciprocal relationships, they aimed to foster a supportive and positive work atmosphere. This was evidenced by the following account:

#### Theme 7: Self-undermining

##### 7.1 Perceived conflict

*“I just feel like it’s unfair and when you do try to stand up for yourself it’s a double-edged sword. You do get what you want, you signed up for it, and you show it’s an unfair roster but at the same time, it leaves a bad taste in your consultants, like why is she complaining about this roster planner who’s a very good doctor in their eyes”* (Participant 17, Medical Officer).

JDs experiencing strain from imbalances in job demands and resources may escalate issues and engage in conflict with peers. In doing so, JDs often encountered personal issues with colleagues or supervisors stemming from perceived task mismatches compared to their peers. In these cases, JDs found it challenging to escalate such issues to peers and supervisors, especially if it involved a fellow colleague. This conflict and attempt to address perceived injustices can lead to self-undermining behavior, as standing up for themselves may inadvertently damage their professional relationships and standing within the team. The struggle to balance fairness in workload and other job demands with maintaining professional relationships underscores the complex dynamics within the healthcare work environment and illustrates how self-undermining behaviors can increase job demands and strain.

##### 7.2 Burnout

*“So, it affects your ability to function not just in the workplace, but outside of it. It affects your mood, it affects your daily motivation, and it is to a degree I think disproportionate compared to just being tired, lethargic kind of. So, a degree that is I think disruptive to a person’s life and function.”* (Participant 12, Resident)

Burnout, characterized by chronic stress, emotional exhaustion, and decreased motivation, is a significant form of self-undermining behavior which affects the ability to be engaged and perform at work. This ongoing stress affects mood, daily motivation, and overall functionality, leading to decreased job performance and increased strain. The disruptive nature of burnout not only hinders immediate productivity but also perpetuates a state of ongoing stress and inefficiency, thereby increasing job demands and further undermining personal well-being.

*“I think at times also where I feel that in my mind, I’m starting to like to dehumanise my patients in a way like they become like tasks to finish so that I can go home. You know, like tasks or barriers standing between me and going home, and I think like sometimes that makes me less and like I guess not as loving as I should be. Yeah, so I feel like that that to be is burnout as well.”* (Participant 3, Medical Officer)

Beyond work performance, some JDs also experienced states of apathy where many of their tasks became monotonous, losing interest in patient care as well as longer-term aspirations about furthering their career. The loss of motivation or interest in these areas which form a significant component of the healthcare profession may cause further distress to JDs through guilt or self-doubt, further increasing strain and impacting their motivation.

#### Theme 8: Perceived job performance

*“So, despite the long hours and everything, it felt very protected, I felt that I learned a lot. And the level of camaraderie I had there definitely compensated for the things that I did not get used to yet. And I contrast that to say, a different posting…, which also had long hours but there was no camaraderie and there was no sense of being in the team.”* (Participant 12, Resident)

Ultimately, the job satisfaction reported by JDs was a nuanced and dynamic state, heavily influenced by their specific postings and a myriad of work-related factors. Particularly in cases where they faced long working hours, the necessity of adequate support for personal and professional growth was further emphasized. A significant challenge lay in the systemic factors that demanded continual commitment and sacrifice from supervisors and peers to maintain high levels of work engagement and subsequent job satisfaction. This highlights the importance of a supportive work environment and robust organizational structures to sustain JD motivation and well-being in the demanding healthcare landscape.

*“I feel appreciated enough but I also feel that there is a lot of work satisfaction just from the nature of the field itself.”* (Participant 16, Medical Officer)

JDs reported a baseline level of job satisfaction derived from the intrinsic nature of their roles as healthcare professionals. This satisfaction stems from the multifaceted aspects of their job, including diagnosing and treating patients, providing care and comfort to patients and their families, and collaborating with fast-paced healthcare teams. The cognitively challenging nature of the profession appeals to JDs seeking personal growth and development. This inherent satisfaction underscores the importance of the nature of the work itself as a significant contributor to job satisfaction within the JD-R framework.

*“Clearly, they are not getting the best of my performances, because I’m getting very stressed all the time. And clearly, they do not seem to want to consider what my feedback is, so I feel undervalued as well. So, it almost feels like wanting to sever a very dysfunctional relationship.”* (Participant 12, Resident)

JDs who expressed dissatisfaction with their work reported that this significantly impacted their motivation and willingness to strive for high performance. The perceived lack of job resources and unaddressed feedback contributed to considerable strain, leading to a work environment where JDs were more likely to commit only to the minimum required tasks, thereby affecting overall job performance.

## 4 Discussion

### 4.1 Summary of findings

**Figure 1:**
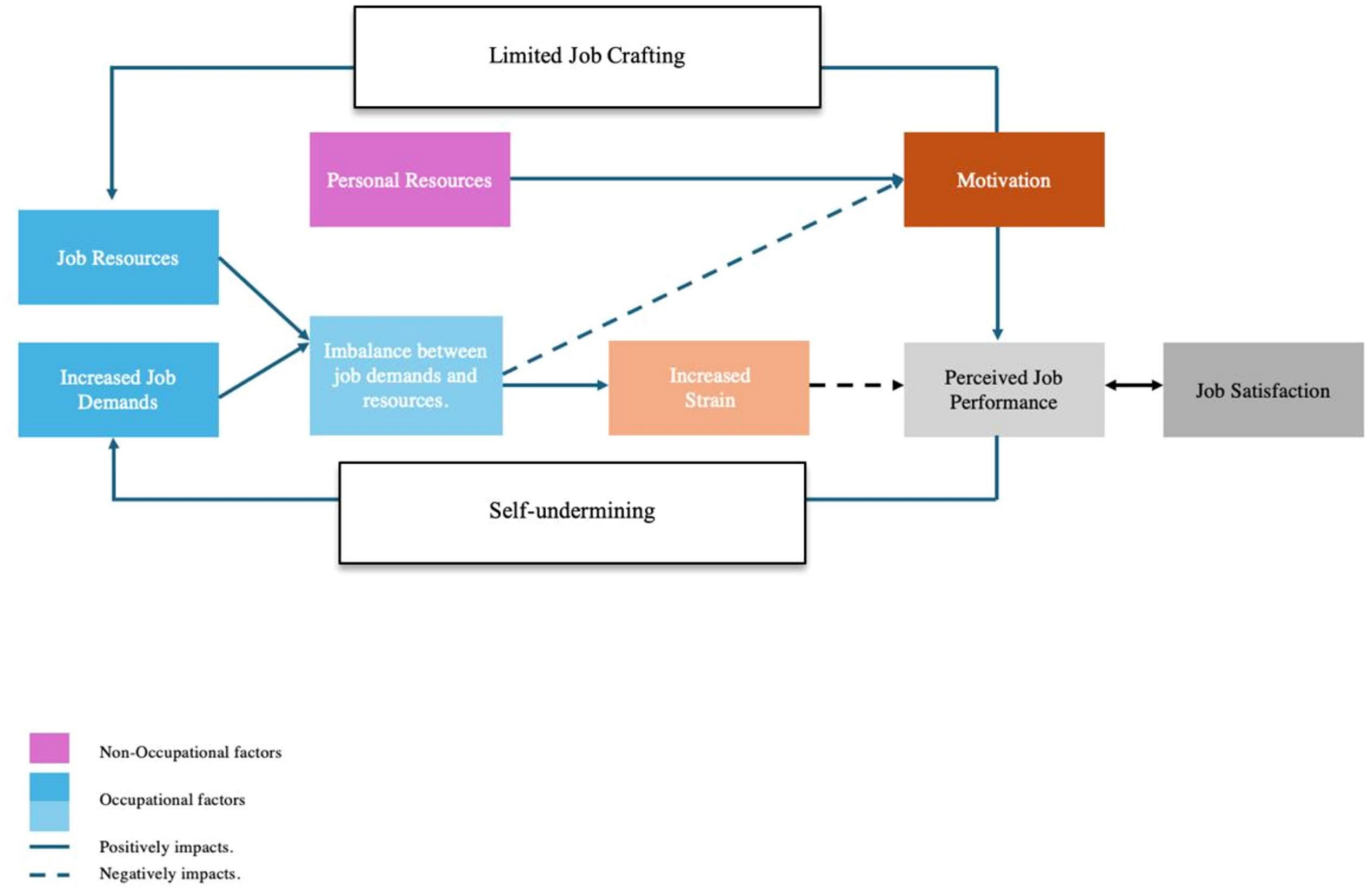
Job-demands resources model for Singaporean JDs.

The study investigates the interplay between various factors influencing perceived job performance and satisfaction among JDs in Singapore. It examines the impact of increased job demands and the lack of adequate job resources, highlighting how this imbalance can lead to strain among JDs and impact patient care. Additionally, it explores how personal resources, such as perceived capability and optimism, act as sources of motivation, modulating the relationship between strain and perceived job performance (Figure 1).

Furthermore, it examines the reciprocal relationship between perceived job performance and satisfaction, emphasising how higher levels of perceived job performance often correspond to greater job satisfaction, and vice versa. An important aspect of this study is the examination of self-undermining behaviors, where JDs may unintentionally create obstacles in their work environment that increase job demands and reduce performance. This may be exacerbated by the issue of limited job crafting, referring to the constrained ability of JDs to modify their job roles and responsibilities to better fit their skills and interests, which can impact their perceived performance and satisfaction negatively.

Job satisfaction and its role within the Job Demands-Resources (JD-R) framework have been validated in previous studies involving postdoctoral fellows and healthcare workers, indicating that the JD-R model effectively addresses job satisfaction (Zhang and Duan, 2023; Jing et al., 2023). In our study, assessing job performance posed challenges due to the nature of qualitative interview where JDs may be concerned about discussing the potential implications of poor job performance on patient care. Regardless, JDs provided rich insights on the demands and resources that impede their overall motivation and ability to flourish at work, including long working hours.

### 4.2 Systemic factors affecting job demands

The International Labour Organization (ILO) defines the maximum standard working time of 48 h per week and 8 h per day as an international norm, JDs in Singapore follow the Accreditation Council for Graduate Medical Education which sets a maximum duty hours of 80 h per week, exceeding ILO recommendations (International Labour Standards on Working Time, n.d.; Safety Institute of Medicine, 2009). Most reported that the long working hours left them little time for self-care or other recuperative actions outside of work and likely exacerbated strain, which are consistent with other qualitative studies conducted in JDs (Petrie et al., 2021; Degen et al., 2014; Hunter et al., 2022). Our findings suggest that excessive strain leads to a reduction in job performance directly through fatigue, poorer well-being and indirectly through self-undermining behaviors (Claes et al., 2023), which diminishes the ability and motivation of JDs to care for patients.

Training and developmental opportunities also pose significant stress on JDs who undertake additional non-work-related tasks such as research and postgraduate examinations to compete for limited specialist training positions. Our findings are consistent with a previous study by Smith et al. demonstrated that uncertainties with specialist training had been a point of concern for doctors in the NHS who intended to leave (Smith et al., 2018), however, the impact of these non-work-related tasks on the job performance or well-being of JDs have not been well-explored. While efforts are ongoing in Singapore to address working hours in JDs, we recommend evaluating the impact of non-work-related tasks and administrative duties within the context of job performance and well-being.

### 4.3 Limited job crafting impacts JDs ability to improve job resources

In a typical workplace setting, job crafting enables employees to address task or relational challenges to improve their job demands and resource balance (Petrou et al., 2018). JDs reported constraints that limit their ability to engage in job crafting. These constraints include the hierarchical nature of the medical profession, mandatory service bonds, and a perceived lack of organizational support for empowering job crafting. Based on previous studies reviewing the four dimensions of job crafting (increasing job resources, increasing social job resources, increasing challenge job demands, and decreasing hindrance job demands) by Tims (2009), our findings suggest that JDs face the greatest challenges with efforts to decrease undesirable work demands as well as increasing work resources. A sustained absence of job crafting opportunities may have implications that extend beyond individual JDs work engagement to include peer job-crafting behavior as well as work engagement (Bakker, 2018), potentially reducing job performance and the quality of patient care.

### 4.4 Impact of work culture on job satisfaction

Work cultural issues voiced by JDs, such as hierarchy, the practice of pre-rounding and the perceived lack of support for junior doctor’s echo concerns in other healthcare systems (Degen et al., 2014). Notably, the lack of organizational support is a common perception shared by JDs in the United Kingdom, Australia and Germany (Krishnan et al., 2022; Hunter et al., 2022; Degen et al., 2014). However, local JDs with experience who had prior working experience in the UK highlighted that the Singapore work culture was more demanding. They expressed heightened work demands due to practices such as pre-rounding, and identified hierarchical structures that hinder effective communication, consequently depleting their job resources. Previous studies have also demonstrated that work cultures emphasising productivity and performance may negatively affect work engagement and consequently impact patient care (Rollins et al., 2021). Similarly, JDs perceived these work cultural factors as hindrances and inefficiencies affecting their ability to communicate effectively with superiors to deliver patient-centred care. These challenges also potentially undermining their capacity for job-crafting, contributing to a negative cycle of increased strain, diminished job performance, and reduced job satisfaction.

The prevailing national culture significantly impacts the quantitative assessment of job satisfaction within a given country, adding complexity to the evaluation of job satisfaction among JDs (Eskildsen et al., 2010) in different countries. Future organizational efforts to monitor job satisfaction of JDs as a means of evaluating the effectiveness of interventions for JD’s well-being should focus on evaluating job satisfaction trends over time, and not comparing job satisfaction of JDs across different countries. Organizations should balance their needs to maintain performance-based cultural practices without detracting from patient care. Enhancing workplace cultures by promoting clear, two-way communication between senior and junior staff will improve access to information, provide valuable feedback, and improve understanding of efforts to address working conditions. Addressing these organizational and support challenges is crucial for creating a positive and supportive work environment that enhances job crafting and improves engagement for JDs in Singapore.

### 4.5 Balancing patient care with fatigue

Job demands, including prolonged working hours and high cognitive and emotional loads, may exacerbate fatigue among JDs (Tucker et al., 2010), leading to increased strain. This is particularly concerning during overnight duties, where the effects of fatigue on patient care are pronounced (Brown et al., 2010). JDs reported significant stress and anxiety over balancing patient care with their own fatigue, often leading to the deferral of non-emergent clinical duties, reducing job performance. This balancing act introduces additional stress and concerns regarding the impact of their decision on patient safety, as well as undermining their professional reputation with other healthcare workers should their omissions result in negative clinical outcomes, possibly affecting their job satisfaction. Our findings highlight the importance of considering these dynamics within the Healthy Healthcare framework, as defined by de Lange et al. (2020), This interdisciplinary, system-based perspective comprises three core pillars: quality of patient care, worker health and well-being, and the organization and practices of healthcare organizations. This framework underscores the necessity of balancing these pillars, recognising that improvements in one area—such as worker well-being or organizational practices—must be managed carefully to avoid potential adverse effects on patient care quality (de Lange et al., 2020). Moreover, the Healthy Healthcare paradigm emphasises a context-sensitive approach, acknowledging that a one-size-fits-all solution is inadequate given the diverse contexts and needs of the healthcare workforce. Therefore, targeted strategies are necessary to mitigate fatigue and enhance JDs’ well-being, in alignment with this system-based perspective, to improve patient care outcomes (Scheepers et al., 2015; Tung et al., 2023).

Chronic work-related stress or burnout may have broader impact on the healthcare profession beyond JDs to patient care (Mangory et al., 2021; Tan et al., 2020; Tan et al., 2022). Other studies have also studied the impact of the COVID-19 pandemic on mental well-being amongst healthcare workers and there is a need to provide robust support to improve working conditions and job satisfaction amongst JDs in order to retain them within the public healthcare system (Alrawashdeh et al., 2021; B. Y. Q. Tan et al., 2020)^.^

### 4.6 Job satisfaction and psychological well-being

Previous studies have extensively explored the relationship between job satisfaction and psychological well-being, underscoring the pivotal role that job satisfaction plays in physician well-being (Capone et al., 2022). Research supporting the Happy-Productive Worker Hypothesis further emphasises the critical impact of job satisfaction and workplace well-being on overall job performance (Salas-Vallina et al., 2020). This aligns with our study findings where JDs with lower job satisfaction reported lower work engagement and greater stress and burnout. Future research should investigate the influence of job satisfaction and other job-related factors on the psychological well-being of JDs.

### 4.7 Determinants of physical well-being to support healthy healthcare

The well-being of healthcare workers, including JDs, is not solely influenced by work-related challenges and organizational factors. Diet choices and exercise are also an important modulator of their overall well-being (Ahmad et al., 2015) which can enhance their personal resources. Similar to other studies, JDs in Singapore often work long hours and face high levels of stress, which can lead to irregular eating patterns, reliance on convenience foods, and limited opportunities for physical activity (de Lira et al., 2023; Mota et al., 2013). Poorer lifestyle choices have been postulated to share a complex relationship with factors such as physical health, cognition, and mental well-being (Bremner et al., 2020; Da Silva et al., 2012; Schultchen et al., 2019), a suboptimal lifestyle pattern will therefore reduce JDs personal resources and their ability to mediate between job demands and their work motivation, negatively contributing to their job performance. This, in turn, exacerbates the challenges faced by JDs. Our study contributes to understanding the complex relationship between worker well-being and patient outcomes. However, further research is essential to explore these interactions in greater depth and to develop targeted strategies that support JDs effectively, while maintaining a balance between worker well-being and patient care outcomes within the Healthy Healthcare framework.

Our study also demonstrated that lifestyle choices such as physical activity or junk food consumption may serve to act as modifiers for work-related strain. To improve the personal resources of JDs, accessible and targeted lifestyle interventions may include initiatives such as providing nutritious meal options in hospital cafeterias, offering workplace wellness programs that encourage physical activity, and promoting education and awareness about the importance of a balanced diet and regular exercise (Cohen et al., 2023; Allan et al., 2017).

### 4.8 Implications for practice and research

Many JDs highlighted their desire to learn and progress in the medical profession as a means to self-actualisation, a concept that may be central to their identity. This aligns with the idea of personal demands (Bakker and Demerouti, 2017), defined as “*the requirements that individuals set for their own performance and behaviour that force them to invest effort in their work, thus associated with physical and psychological costs*” (Bakker and Demerouti, 2017). Although personal demands have been proposed, they have yet to be formally included in the JD-R model. Our findings suggest that the definition of personal demands could be expanded to include individual requirements in self-care, faith, family, social, physical activity and social areas. We believe that, beyond JDs, individual workers have specific needs that, when met, enhance their desire to set higher performance and behavior standards at work.

## 5 Limitations

Firstly, the absence of member checking, a process where participants are given the opportunity to review and validate the interpretations of their experiences, poses a limitation. Integrating member checking could potentially enhance the validity of our findings by enabling participants to confirm or provide feedback on the interpretations of their experiences. Secondly, the utilization of a deductive framework analysis may not exhaustively uncover all potential factors influencing job performance among JDs in Singapore. This limitation underscores the necessity for additional inductive exploration in future studies to comprehensively understand the multifaceted aspects of job performance in this context.

## 6 Conclusion

The study highlights an imbalance of work and personal demands and resources on Singaporean JDs based on the JD-R model, tilting the balance negatively and potentially impacting job performance. Holistic, systemic efforts may be needed to alleviate this balance while enabling JDs to implement job crafting. Effective measures include reducing working hours, improving communication, and ensuring a safe working environment. A layered combination of organizational support, lifestyle interventions can foster a supportive environment for JDs and other healthcare workers. Ultimately, these efforts will contribute to improving the quality of care provided to patients and enhancing the overall sustainability of the healthcare system.

## Data Availability

The datasets presented in this article are not readily available because participants did not provide consent for sharing of the dataset. Requests to access the datasets should be directed to chuajialong@gmail.com.
