# Supplementary material for: In the shoes of junior doctors: a qualitative exploration of job performance using the job-demands resources model
Source: Front Psychol. 2024 Oct 24;15:1412090. doi: 10.3389/fpsyg.2024.1412090 (PMC11540654; doi:10.3389/fpsyg.2024.1412090)
Supplement: Supplementary file 1 [file Table_1.DOCX]

**Appendix A: Interview Guide**

Participant Interview Guide

Objectives

1. Explore lived experiences of junior doctors based on the JD-R model
   1. Job demands
   2. Job Resources
   3. Personal Resources
   4. Motivations
   5. Strain
   6. Job-crafting
   7. Self-undermining
   8. Job Performance
   9. Work Satisfaction
2. Assess current level of physical well-being
   1. Learn more about dietary and physical activity practices of JDs
   2. Understanding the knowledge, attitudes and barriers for healthy living

| **Introduction:** |
| --- |
| Hi, thanks for agreeing to speak to me about your working life a junior doctor. This interview will take about 30-45 minutes of your time and will include general as well as specific questions about personal and occupational and how they may affect you at work. For reference, I will be taking some notes to record my impressions during the interview, and I will also need to make an audio recording of this interview which will only be used by the research team for transcription, you will be assigned an interview number and no other personal identifiers will be recorded.  Some aspects of the interview may cause you to be emotional or upset and that’s OK, if you would like me to stop recording, please let me know, if I sense that you’re reacting negatively, I will also indicate to you that I will be switching off the recording as well. If at any time you would like to terminate the interview, or if I sense that this process is causing you unnecessary distress, I may also terminate the interview. |
| **General questions** |
| 1. Biodata    1. Age    2. Gender    3. Education (medical school local/overseas)    4. Work experience (posting locations)    5. Current appointment (HO/MO/Junior resident/Senior resident/Staff registrar)    6. Current posting 2. General question    1. In the next 3-5 minutes, tell me more about your experience as a doctor, any topic that you wish to share.    2. How long have you been working and how has the experience been?    3. If there is one thing you would want to share about your experience as a doctor, what would it be? |
| **Job Demands:** Job demands necessitates the continual application of physical, cognitive, and emotional efforts or skills resulting in the psychological, cognitive or physical consequence |
| Open-ended  Can you describe a typical workday for you?  Possible Prompts   - What are your usual working hours - What aspects of your job do you find challenging or stressful - Are there specific tasks that are particularly challenging - What strategies do you use to cope with work and some of the stress that may bring? - Has there been situations in the past at work where you felt overwhelmed? |
| **Job Resources:** Job resources encompass the physical, social, financial or organizational aspects of a job that aid in achieving work-related outcomes while mitigating the impact of job demands on individuals. A deficiency in resources to address heightened demands can lead to a demand-resource imbalance. |
| Open-ended   1. Describe what your work environment is usually like? 2. What resources or support do you have at work to help manage your workload?   Possible Prompts   - How would you perceive the availability or adequacy of these resources? - How would you describe your access to administrative support? - What training or mentoring resources do you have to help manage your work? - Can you describe any positive experiences where work forces helped you to manage your work? - How did these resources affect your work and well-being? - Are there any resources that you feel are lacking or inadequate? - Can you describe any negative experiences where work resources were inadequate in helping you manage your work? - What are your thoughts about your salary as a JD? - Are there any organisational programmes that your hospital has initiated to help your well-being? How has it been? |
| **Personal Resources:** Personal resources encompass the perceived ability to fulfil work duties and achieve outcomes, organizational-based self-esteem fostering a sense of value within the organization, and optimism nurturing a positive outlook. These factors enhance motivation, positively impacting work engagement, and mitigating the adverse effects of strain on work engagement. |
| Open-ended   1. How would you describe your physical and mental well-being 2. How do you maintain your physical and mental well-being outside of work   Possible Prompts   - Do you engage in any activities or practices to stay healthy and resilient - How are you balancing your work-life commitments? - What strategies do you use to ensure that you have time for both - How do these personal factors help you to manage your workload? - Outline a positive experience where your personal resources helped you to cope with a challenging situation at work. - Do you think you spend enough time on your life commitments? Why or why not? |
| **Motivation:** Motivation drives workers to be goal-oriented and focused on tasks. Job resources, both intrinsic and extrinsic, enhance work engagement, promote exceptional performance, and facilitate goal achievement. Whereas, personal resources, intrinsic to individuals, positively motivate and impact work engagement by instilling a sense of fulfilment and importance in their tasks, aligning with work goals |
| Open-ended   1. Do you feel appreciated at work? 2. What motivates you as a junior doctor?   Possible Prompts   - What are your aspirations and what do you hope to achieve - Has your motivations changed as a medical student to a JD? - Are there specific examples you recall where someone has shown appreciation for the work that you have done? - Are there examples as well where you felt a lack of appreciation? |
| **Job Crafting:** Job crafting refers to the process where employees actively make changes in their work tasks, working relationships and perception of work to make it more meaningful. |
| Open-ended   1. Have you made any changes to your job tasks, relationships, or perceptions to make your work more meaningful or manageable? 2. How do you interact with your colleagues and supervisors to improve your work environment?   Possible Prompts   - Can you give examples of how you have crafted your job to better suit your needs and preferences? - Are there specific actions you take to build positive working relationships? - Were there times when others had tried to feedback or drive changes in their working environment or tasks? What happened? |
| **Self-Undermining:** Self-undermining refers to behaviours that create obstacles, which may lead to higher levels of job demands and even higher levels of job strain. |
| Open-ended   1. Have you encountered situations where your actions or decisions inadvertently made your job more difficult? 2. How do you address conflicts or challenges with colleagues or supervisors?   Possible Prompts   - Can you give examples of how you have crafted your job to better suit your needs and preferences? - Are there specific actions you take to build positive working relationships? |
| **Strain:** Strain is defined as negative consequences that arise due to the imbalance between job demands and resources. Strain can be further classified into job and non-occupational strain. |
| Open-ended   1. Have you experienced any physical or emotional strain as a result of your job? 2. How do you address conflicts or challenges with colleagues or supervisors?   Possible Prompts   - Can you give examples of how you have crafted your job to better suit your needs and preferences? - Are there specific actions you take to build positive working relationships? |
| **Job Performance / Work Satisfaction:** Satisfaction, or job satisfaction, indicates employees' level of contentment with their job. This satisfaction is closely linked to job performance, as higher satisfaction often indicates that employees are more committed and demonstrate higher vigour at work. |
| Open-ended   1. Do you feel satisfied working as a junior doctor? Could you expand on your rationale 2. How are you coping as a junior doctor?   Possible Prompts   1. Do you feel that you get sufficient social interaction at work and outside of work? Why or why not? (explore whether subject is receiving sufficient belongingness and love needs, social isolation? 2. Do you feel that your current experience as a junior doctor is beneficial in you reaching your fullest potential? Extending beyond a doctor to you as a person. 3. If you could change one aspect of your working life as a junior doctor, what would it be? 4. Have you ever encountered situations when you felt unfairly treated? If not, have you heard stories about friends or colleagues being treated unfairly. How did this make you feel? How did this impact you work, sleep or social life? 5. Do you feel that your renumeration is sufficient? Do you feel that an increase in pay would increase your work satisfaction 6. Comparing to the perceptions you had of junior doctor life in Medical School, how has your perception changed since joining the workforce? |
| **Questions about Job-crafting, about previous efforts to advocate or provide feedback on working conditions** |
| Possible Prompts   1. What are some of your thoughts about working life as a junior doctor in Singapore? How would you improve the working conditions? 2. Do you have any experience or have you come across other individuals who have tried to suggest changes for improving the working environment? What was the outcome? 3. (For more experienced junior doctors) In your view, what is it like starting you as a junior doctor today and how has it changed over time? |
| **Additional Questions** |
| **Diet**   - Tell me about your daily meal routine. - Enablers to good dietary practices:   1. Do you feel that your occupation incalculates good dietary practices? Why or why not?   2. What steps have you taken to improve your dietary habits in the past? How did you think you fared? - Barriers to good dietary practices   What are some unhealthy dietary choices that you’re making currently? What are some factors that lead to these choices?  **Exercise**  Open ended  What is your usual physical activity routine?  Possible Prompts   1. Do you know of any recommendations regarding sufficient exercise? 2. How much exercise do you get every week? 3. In your opinion, are you getting sufficient exercise? 4. Enablers to exercise    - 1. What factors in your life enabler to adopt better exercise patterns? 5. Barriers to exercise   Open-ended  What has your sleep been like as a student compared to working as a doctor?  Possible Prompts   - 1. What are some good sleep hygiene practices?   2. Do you know of the recommended duration for daily sleep?   3. How much do you sleep a day on average?   4. What are some barriers to you achieving good or sufficient sleep?      1. Why do you feel that you’re able or not able to achieve sufficient sleep?      2. What is the impact of work as a junior doctor on sleep?      3. In the past week, have you had sufficient sleep?   5. What are some enablers to you achieving good or sufficient sleep? |
| **Closing Questions** |
| Given all we’ve discussed today, do you happen to have any other experiences or thoughts that you would like to share?  Thank you very much for your time, this will help us to better understand the lived experiences of Junior Doctors in Singapore |
