# Supplementary material for: In the shoes of junior doctors: a qualitative exploration of job performance using the job-demands resources model
Source: Front Psychol. 2024 Oct 24;15:1412090. doi: 10.3389/fpsyg.2024.1412090 (PMC11540654; doi:10.3389/fpsyg.2024.1412090)
Supplement: Supplementary file 2 [file Table_2.DOCX]

| Theme | Sub-theme | Supporting Quotes |
| --- | --- | --- |
| 1: Job Resources | Lack of adequate manpower to cope with increased demand | *“The manpower for the different teams is very low, with about one to two HOs maximum per team … the main challenge that we face is the posting was having a consistent shortage of manpower, which made for very long working hours, and a lot of pressure on each individual, especially having to cover for others while they're post call and then taking care of your own patients as well.” (Participant 10, House Officer)*    *“It becomes very stressful when there's lack of manpower because I think those were the times where I thought I was most stressed … because there are so many patients for me to take care of, it’s not that I can't take care of all them but like, you can't give them to me all at the same time.”*  *(Participant 6, Medical Officer)*  *“I think the reality is the departments themselves can request for whatever amount of manpower but … there is a limited amount of manpower at the end of the day. So it's not possible that you can magically conjure up more manpower to give to this department” (Participant 13, Senior Resident)*    *“Because there are so many constraints in the system I think probably the only thing that can be done in terms of communication wise from the superiors to the juniors, that being all these things are there, the constraints, you know you’re putting a tight spot, but communication can help things run. Unlike a boss who assumes that you are just doing your duty just as you're paid to do so … a boss who assumes that you are replaceable…” (Participant 5, Medical Officer)*  *“I know they need MOs because two MOs had already taken no pay leave in the middle of that posting, because they just couldn't handle it anymore. ... I just feel like it's unfair and when you do try to stand up for yourself it's a double edged sword… you show it's an unfair roster but at the same time, it leaves a bad taste in your consultants (mouth), like why is she complaining about this roster planner who's a very good doctor in their eyes…” (Participant 17, Medical Officer)* |
|  |  |  |
|  | Lack of effective Organisational initiatives to cope with increased demand or its consequences. | *“I think not having a proper feedback channel within the employment makes a very big difference for me because if I knew that I could give feedback … and I knew that something would be done from that, I would have been more willing to bear with it ... But ultimately, if I feel like, no matter how high I escalate the issues, that if I tried to give constructive feedback … everything just falls on deaf ears and so I’m giving feedback, then I don't see what role I can play in that workplace anymore.”(Participant 4, Medical Officer)*  *“I mean, there's a lot of things to help with wellness but honestly, I think for the doctors, at least, we don't really have time to attend all these things, I mean it’s more for other admin people (administrators) because I think the way my shift works … so our day to day job is like between around 6:30 or 7:00am to finish over about 5 - 6:00pm I don't think anyone wants to stay back after that for this mindfulness class or yoga class, when I could be doing other things outside.”*  *(Participant 14, Medical Office(* |
|  | Hierarchical Nature of the healthcare profession | *“It's very different from Ireland (alma mater), so I think in Singapore the culture shock … (comes from) this clear hierarchy of consultants, ACs (associate consultants), Registrars, MOs and more HOs around it. Yeah, it's a bit of a shock to me that the consultant is this king that walks through the wards and you present the cases as they walk by.” (Participant 17, Medical Officer)*  “Again, this is all hierarchical, and it is the some of the consultants, especially the older ones, don't really know how their words affect people and I have heard some really terrible things being said to juniors, which is not going to help their wellbeing, not going to flatten the hierarchy, and not going to boost morale. It's very condescending, like people are being told, ‘how did you pass your exams, I need to go and speak to the exam board and think about taking you back’. You know, I mean, like, it's just not helpful and them saying, ‘oh, which medical school did you go to?’ And criticizing you like, that's just not helpful, it's, again, not going to help wellbeing because you're basically creating this divide and I think it also affects patient safety, because you will be worried about speaking up to your consultants, because you'd be worried that they're going to be seeing some hurtful personal things like personal attacks.” (Participant 20, House Officer)  “There some instances where like, because there's so many in the hierarchy … has house officer, medical officer and registrar and Associate Consultant and consultant and everything somewhere along the line, some misunderstandings come to play, and then more often than not, they will blame … the house officer but because you're the most junior . And the house officer won’t try to say that ‘actually someone told me something otherwise, or actually this person said this’, you just keep quiet and suck it up.” (Participant 6, Medical Officer)  And then I guess the second thing would then be, certain team dynamics. So if you feel like you're not so comfortable in a team, or when let's say some of the seniors in the team sometimes your work styles don't get along or sometimes if they are a bit more anxious, sometimes they will also tend to be a bit more I would say overbearing sometimes so then that will lead to, I guess, more pressure and probably a tougher environment. (Participant 8, House Officer) |
|  | Lack of Training Opportunities | *“I'm aiming to do a residency, so I think it's particularly stressful because you feel the constant need to perform well at work. Yeah, to like impress people and like not mess up because like especially within like smaller departments like even one mess up then like people there can be a lot of like talk behind your back and like bad impression can be formed, so I think it's just that constant stress of having to perform all the time.”* (Participant 3, Medical Officer) |
|  | Supportive Actions from colleagues / supervisors | *“When I was a HO … the MO would come down to me and say “you should sleep for one hour then I (will take turns and) sleep for one hour. We don’t always have to both be up.” …. Then when I was an MO, when I had a choice I would try to chase them away to sleep for an hour… even an hour of sleep can be the difference between being absolutely miserable and just being mostly miserable” (Participant 7, Medical Officer)*  *“Support that you get at work is a very important aspect…*  *You know if you are very, very busy then they (peers) will step in to do things for you. They will take over some of the tasks. Like in medicine, usually you split the work by different patients, so for e.g.I take care of patient ABC, then you take care of DEF.*  *Yeah, but then you know when when it gets down to it your friend will help you also, so I think I felt supported. I felt supported by that many times.” (Participant 1, Medical Officer)*  *“I think my experience with good bosses, were when they stepped down in terms of like helping with certain patients that they know very well, sometimes when you have a sick patient, they will also step down and help, which I find very impressive and a few guys, when they come down to our level, not all the time, but sometimes or when they understand that we have a lot of patients to deal with, they are able to understand that we may not be able to fulfill everything all the time on time.” (Participant 10, House Officer)* |
| 2: Personal Resources | Perceived capabilities to fulfil responsibilities | *"I think to some extent, it does, because with experience you kind of know what common mistakes you've made, and you know what to look out for. If you know you’re tired and you know, you're doing something where you might make a mistake, I think, in a sense, you can kind of remind yourself, consciously to look out for the mistake that you usually make when you’re tired. But I think you are still as prone to errors, it's just that your checking system is a bit better with experience."* (Participant 11, House Officer)  *Personally, I think I manage it well, because I take a lot of initiative to take care of my mental health with no help whatsoever from the system, like I go for therapy, I'm driving back from therapy right now which really* *helps to have like, a place where you can talk about all the things you can control and then talk about like, okay, so what can I control, what are the things that I can do to like, help me find joy on these that I am feeling like stress overwhelmed,* (Participant 16, Medical officer)  *I* *have a routine that essentially to before I leave work to sort of disconnect from what essentially and also have a routine before I go to bed forget about the day and then go to bed so then I don't think about things that happened during work. So suppose it helps that obviously it hasn't really helped with my time management and managing stress* (Participant 9, House Officer) |
|  | Innate Optimism or personal demands | *But at least for me, I don't feel that's the case, I think there's more about things in life than excelling just at work. I think, building yourself as a person is more important. So, I guess it's really all about priorities but some people of course, really see work as everything so I’m sure for those people things will be a lot more difficult, because you will then expect yourself to be at your best all the time*. (Participant 14, Medical Officer)  ***"****I think I would I don’t really face that much stress on the personal or social side of things. I think one of the main factors that help to reduce stress, personal and social life itself, or being able to survive on this since I think that's a very big psychological safety factor."* (Participant 10, House Officer)  *I think it's probably a dual thing of number one, don't expect to know everything because the moment you put that sort of expectation on yourself, you're going to end up burning yourself out, you'll always find someone who's better than you in knowledge and one is better you in skills which is another way of saying no matter how busy the posting is you could have a chill one, it could be a busy one, find time to relax, find time to do your own thing so that you can also recuperate, it's not always about trying to be the absolute best."* (Participant 12, Medical Officer) |
| 3: Job Demands | Long Working Hours | *“So I mean, from a general perspective,... the working hours, on average, about 12 hours a day … On top of that, we will have to work on weekends… On top of that, we still have calls …that immediately adds like 30 hours and you can do like maximum three calls per week. So that can add up to like 100 total (working hours per week) already.”*  *(Participant 4, Medical Officer)*  ***“****I will hear from all my seniors that are here that working life in Singapore would be hard… yes sure sometimes hours are long, but it's not anything that is, I guess extreme in some ways, apart from a few select teams, but I do feel the two main things that will lead to feeling a lot more tired or burnt out, the first one is calls because everybody hates calls, and it’s 30 plus hours of nonstop work”*  *(Participant 8, House Officer)* |
|  | Increased Emotional Load | *“I realized that you're kind of in a position where … you're just basically balancing expectations from different people, like your consultant, then your seniors, and then the nurses and patients and patients families which is just not something that medical school really prepares you for.” (Participant 2, Medical Officer)*  *“So if they are not ready to deal with the breadth of the conditions, so they are not ready to deal with one O&G patient walking in requiring VE (vaginal examination) and speculum and then the next patient is like a baby, like two months old with jaundice, and then the next patient is like some geriatric patient just fell down, and then the next patient is like poorly control diabetes… if you haven't done Polyclinic posting before, and you haven't done a breadth of hospital postings, you're like, I've done like three years of GS and ED postings, and then that's not going to be quite enough in terms of your training and your preparation might not be too good. Yeah, so I guess personal capacity matters and then the second thing is that confidence to deal with multiple conditions in a short duration of time and that keeps changing with every patient.” (Participant 5, Medical Officer)* |
|  | Increased cognitive load (pre-rounding, administrative tasks and high professional standards) | *“I think pre-rounding is okay when they speed things up but to expect so much from the pre-rounding is a bit much when they quiz you about the labs (laboratory results) trending over three days and you can't remember every single patient’s (results).” (Participant 17, Medical Officer)*  *“I remember you know coming (for) rounds at 6:00 in the morning so if you have to reach by say 5:30 to pre round and I mean officially you’re not supposed to but let’s be honest, it happened and it happens.” (Participant 18, Medical Officer)*  *“It's very interesting because the management seems to think that pre rounds are optional … But that's not true because what we do is that we consolidate all the information … past medical history issues active, latest investigations, latest findings, and we consolidate all this together presented to the consultant who then makes a judgment on the plan. which is why we think that pre-rounds are so integral to get everything together in a nice package without missing anything, so that we can get the patient on the correct plan for the day.” (Participant 4, Medical Officer)*  *“My boss was saying that …. when we go into medicine, we are … expected to be the first one in, and the last one out … he's trying to say we shouldn't get upset when …  different standards are applied to us because as health care workers we kind of signed up for it.” (Participant 2, Medical Officer)*    *“I guess I'm I'm also a bit of a perfectionist myself, so I think I self-contribute to my high expectations. So I think surgical specialties … is especially bad (in relation to professional standards) because the training has always been traditionally quite bad. It's almost like a given that you have to suffer for the specialty to prove your prove your dedication to the craft” (Participant 3, Medical officer)*  *“if you have a complicated patient, you then have to speak to your consultants. So as consultants definitely cause stress, if you're calling someone who you don't like or you know is a little bit mean or whatever that was on call the day before they’re stressed because you know that your experiences with them may not be very nice.” (Participant 20, House Officer)*  *“After I made that one mistake, he (consultant)  kept going after, like, all the small details to the point where I feel like, every morning when I present I have to back up what I said, like, Oh, it is here, or I saw this here, and then show it to him so that he doesn't like doubt what I'm saying.” (Participant 11, House Officer)*  *“There are others (consultants) that make 1000 referrals or phone consults and that's very stressful, because when you finish rounding, you have a lot of changes to do and then often run into the time when you are supposed to go for teaching, etc.”*  *(Participant 10, House Officer)*  *“But then apart from that work-related stresses essentially, recently, there's a lot of admin related to work even at the end of day if you go home… basically, you're not being able to use it and work at home, either, it means you’re now spending a lot more time at work, trying to make sure you settle everything you have to do.”*  *(Participant 13, Senior Resident)*  *“Antipathy of the administration things, like don’t pay your call claims for four months, like requiring you to go to teaching at 2:00 PM when you are post call; and rejecting transport claims when technically work day starts at 8:30 is your problem you have to come in at 6:00 o’clock. So these kind of things you just add up after you know four months of being busy I just don’t care if you don’t hire, I will sort something else out.”*  *(Participant 7, Medical Officer)*  *“I also struggle with is having to juggle a lot of different things like attending teaching during lunchtime, which is almost impossible … doing all these accessory things when you are just trying to settle your primary work”* (*Participant*  *10, House Officer*) |
|  |  |  |
| 4: Motivation | Increased Work engagement | *“it's a bit mixed but overall, it's more positive, I genuinely really enjoy being a doctor, I enjoyed the role of what it entails to be a doctor, of what we try to do within a community, I enjoy the roles where I get to interact directly with patients. (participant 12, Medical Officer)*  *“I do feel appreciated at work. I've got thank you cards from patients and staff. So I do from that... Of course I'm appreciated by my superiors as well, because of things that I have done and generally they do enjoy rounding with me with me and I feel very well received especially in (department redacted) and also (department redacted)”* (Participant 15, Medical Officer)  I feel like my faith kind of helps me. Because like I guess I believe in God. Helps me to do. Like translate like there is a purpose and stuff and like you know when bad things happening, then I guess like praying and stuff that help me. Good support network outside of work. (Participant 2, Medical Officer)  “*Working as a junior for a while. You also feel like you are very, very replaceable. And I guess that also like makes you feel... Like your work, I don't know. It's not very meaningful. Well, like you're not very meaningful.”* (Participant 3, Medical Officer)  *“But a big caveat I will say here is that even though I started off in (department redacted), and hours was very long, I had a really good team. So I had fellow MOs, who all helped each other out, I had bosses who didn't raise their voice to anyone, who gave us a lot of teaching. So despite the long hours and everything, it felt very protected, I felt that I learned a lot. (participant 12, Medical Officer) “I know they need MOs because two MOs had already taken no pay leave in the middle of that posting, because they just couldn't handle it anymore. ... I just feel like it's unfair and when you do try to stand up for yourself it's a double edged sword… you show it's an unfair roster but at the same time, it leaves a bad taste in your consultants (mouth), like why is she complaining about this roster planner who's a very good doctor in their eyes…” (Participant 17, Medical Officer)* |
|  | Commitment to learning and professional growth | *“At the end of the day, you have to accept that you're still human and mistakes have to be made and can be made. You have to face the consequences for them, of course, but part of it is realizing that you're never your best all the time … for me least I take it as a learning opportunity” (Participant 1, House Officer)*  ***“****Yeah, I think it varies by point in time or what is sort of going on at work, but I think there's also an element of it, where you sort of recognize the difficult parts of it are just part and parcel of the job and at a certain point when it doesn't feel like there's a whole lot you can do about it doesn't make too much sense to dwell on it either and at this point I do focus a lot more on the positives.” (Participant 12, Medical Officer)* |
| *5: Increased strain on JDs* | Increased Job Strain | *“When I was a HO, one patient’s son made me cry … he was just being very unreasonable and accusing me of stuff that wasn't true, like purposely delaying the discharge or like coming up with reasons to make his mom stay … they (verbally abusive caregivers) also don't get any repercussions for this type of behaviour”. (Participant 3, Medical Officer)*  *“Across the board, people are very exhausted, I’ve only started (house officer) work a couple of months and start to feel tired, you feel like you are inadequately rested, you feel like you don't have adequate personal time to take care of yourself. And then after beyond that, you feel like you don't have enough time to take care for patients, you don't feel that you have the capacity to take care of patients at the level you will want them to be cared for. Beyond that you become very exhausted, you just become like more like a machine that's churning out changes, churning out entries rather than caring for patients. And if I can't even have enough time to have meals to go to the toilet or take care of my family then my priority no longer becomes taking care of the patient instead it comes time to make sure that I get enough time so i can take care of myself and I feel like this is a very prevalent sentiment that most house officers have. There are a couple of exceptions that sometimes do vary but they are minority.*  *(Participant 4, House Officer)* |
|  | Increased non-occupational strain | “*The fact that our timings are very erratic ... It's difficult to … set times on social engagements or even meet your friends or even go home for dinner because you just don't know when you have to stay back ...*” (*Participant 10, Medical Officer*)    *“socializing is very hard to do, especially if your friends are also doctors because everybody also has their own schedules, so that might happen maximum once a month for me. So that is not a reliable option for me to relieve stress.” (Participant 8, House Officer)*  *“For me, doing some kind of exercise is very helpful and once your day ends late, that outlet or that window of time is taken away because when I get home I’m just hungry then after I eat I can’t exercise, then after that, it's too late that’s time to sleep, then you're just like okay, this sucks. Okay, maybe tomorrow. So then in the end, it just becomes weekends or something.” (Participant 18, Medical Officer)* |
| *6. Job Crafting* | Limited Job Crafting | *“There's a lot of things you want to say and can't because we are bonded cannot afford to lose our jobs we cannot afford to lose the money, many of us are still trying to get residency so we can't afford to lose our career. So, You can’t speak up it’s made difficult. So anonymously feedback channels would be great.”*  (Participant 4, Medical Officer)  *“Oh I see the other nurse ask “help me turn the patient?” I say no problem, I help them... I think all these small things do help. So they feel like, okay, this doctor is helping me … And then sometimes the next morning, they'll be like, Oh, hey, you know, I got you coffee, or bread or something.”*  (Participant 18, Medical officer)  *“I think not having a proper feedback channel within the employment makes a very big difference for me because if I knew that I could give feedback … and I knew that something would be done from that, I would have been more willing to bear with it ... But ultimately, if I feel like, no matter how high I escalate the issues, that if I tried to give constructive feedback … everything just falls on deaf ears and so I’m giving feedback, then I don't see what role I can play in that workplace anymore.”(Participant 4, Medical Officer)* |
|  | Relational Crafting | *“Oh I see the other nurse ask “help me turn the patient?” I say no problem, I help them... I think all these small things do help. So they feel like, okay, this doctor is helping me”* ( Participant 18, Medical officer)  So generally, across all my postings, we do try to like as a house officer, now as a medical officer, so as house officer we always make sure that we try to help one another a thing that has been the culture I've been blessed with so far, at least within people the same grade. (Participant 6, Medical Officer) |
| *7: Self-undermining* | Perceived Conflict | *“I just feel like it's unfair and when you do try to stand up for yourself it's a double-edged sword. You do get what you want, you signed up for it, and you show it's an unfair roster but at the same time, it leaves a bad taste in your consultants, like why is she complaining about this roster panel who's a very good doctor in their eyes” (Participant 17, Medical Officer).*  *“I find hospitals here are very reliant on junior manpower. So your patient care is very junior doctor dependent, that's why when I hear people say, Oh, this hospital is bad like the care is lousy that kind of thing… And somehow I understand because sometimes it's not that the hospital is bad. It's not like the consultants are bad but if your consultants can't see everything there are clinics are doing all this stuff, they're busy and so the care lies at of course the junior doctors and if they don't really bother they're just there for more tags or they are there for whatever probably residency but they it’s a chill posting, then you don't really do as much. (Participant 18, Medical Officer)*  *“So it's something that always preoccupies our conversations, oh, when are you getting leave? When are you getting off (work)? Because all we want is just to rest and I think that that is a very big indicator that we are kind of burned out”* (Participant 10, Medical Officer) |
|  | Burnout | *“So it affects your ability to function not just in the workplace, but outside of it. It affects your mood, it affects your daily motivation, and it is to a degree I think disproportionate compared to just being tired, lethargic kind of. So a degree that is actually I think disruptive to a person's life in function.” (Participant 12, Medical Officer)*  *For me, it's working past your own limit to the brink of just giving up but you have this obligation to continue on because the manpower is so bad. There's no you should take MC [unintelligible 00:51:29] because if you take MC, the roster just goes haywire, people who need to stay post call, you feel guilt, wanting to take a rest and that just perpetuates your burnout because you can take a break because you will disrupt someone else's break it is never ending just like go just working yourself to death basically. It's basically what kills your passion to do medicine in the first place, I find. I think that's the best definition of burn out, just killing a passion for medicine and you forget why you went into medical school in the first place. (Participant 17, Medical Officer)*  For me, burnout means it's almost equivalent to depression, I think, in my opinion, where you are numb, you lose a lot of yourself, you are unable to appreciate the things that you used to appreciate so Anhedonia but then also, you're still not able to sleep or get good sleep. So you are anxious. And it's just like a vicious cycle. And it's different from depression in that there is a clear trigger and cause for it and the triggering cause is not equivalent, in every single individual. I can be burnt out by this stressor and you may not be burnt out by this stressor. But you could be in my shoes and not be burnt out and I applaud you for that. But unfortunately, often my resilience, I guess is not to that standard. So, I guess burnout is really when you are pushing so hard that you are just constantly overloaded for a long period of time, which then obviously, affects the other the things that you usually do to balance it, and then it gets to the point where it's a little bit too far. And then you're unable to function. Your memory gets worse, your concentration is worse. So I do, like in it a lot to depression but there is a clear source and once you take out that source it then get better, hopefully.  [When asked if they had experienced burnout] Absolutely. I mean, that's why I'm not taking exams. So I think it definitely was burnout and I'm finding that my burnout threshold is potentially getting less and less like, I've been burnt out before, majorly … was working quite hard I felt a little bit hopeless and I felt really burnt out at that point. And then subsequently, I have felt elements of burnout in Singapore as well but most recently, it was to the point of I can’t continue studying for this exam, I have no concentration, I am miserable. I need to just stop. (Participant 20, House Officer) |
| *8*: Perceived Job Performance |  | *“Clearly, they're not getting the best of my performances, because I'm getting very stressed all the time. And clearly, they don't seem to want to take into account what my feedback is so I feel undervalued as well. So it almost feels like wanting to sever a very dysfunctional relationship.”* (Participant 12, Medical Officer)  *“So despite the long hours and everything, it felt very protected, I felt that I learned a lot. And the level of camaraderie I had there definitely compensated for the things that I didn't get used to yet. And I contrast that to say, a different posting…, which also had long hours but there was no camaraderie and there was no sense of being in the team.”* (Participant 12, Medical Officer)  *“I feel appreciated enough but I also feel that there is a lot of work satisfaction just from the nature of the field itself.”* (Participant 16, Medical Officer)  *“(When asked about their current work satisfaction) That's a loaded question. I think it's loaded because work satisfaction is from so many different things. I think it could always be better. I think I am still being satisfied by work because of the type of work that we do, it's always satisfying when a patient who is in diabetic ketoacidosis and you are able to reverse their metabolic acidosis. It's satisfying in that way.*  *I feel like I could definitely be more remunerated for my job, especially for the number of hours that I work. So that's not satisfying at all. Financially, I'm not satisfied because I'm comparing to other professions and for the amount of work that we have to do in terms of med school postgraduate exams, nights worked, it's not satisfying at all. In terms of Singapore itself again, culture does make it more difficult to feel satisfied. I think culture does promote a lot of community, even when patients often leave family discussions not satisfied.*  *So I guess, working as a junior doctor I can't say that it would be miles different if I was elsewhere but and the grass is always greener on the other side, always. So I just don't know if… I feel also part of it is that culture I talked about before the feeling you feel you that you’re not heard until you are a consultant, senior doctor who's been working for many years.”* (Participant 20, House Officer) |
